# Melatonin Reduces Oxidative Stress Damage Induced by Hydrogen Peroxide in *Saccharomyces cerevisiae*

**DOI:** 10.3389/fmicb.2017.01066

**Published:** 2017-06-15

**Authors:** Jennifer Vázquez, Beatriz González, Verónica Sempere, Albert Mas, María Jesús Torija, Gemma Beltran

**Affiliations:** Departament de Bioquímica i Biotecnologia, Facultat d’Enologia, Universitat Rovira i VirgiliTarragona, Spain

**Keywords:** *Saccharomyces cerevisiae*, melatonin, glutathione, ROS, oxidative stress response, gene expression

## Abstract

Melatonin (*N*-acetyl-5-methoxytryptamine), which is synthesized from tryptophan, is formed during alcoholic fermentation, though its role in yeast is unknown. This study employed *Saccharomyces cerevisiae* as an eukaryote model to evaluate the possible effects of melatonin supplementation on endogenous cellular defense systems by measuring its effects on various cellular targets. Cell viability, intracellular reduced and oxidized glutathione levels (GSH and GSSG, respectively), reactive oxygen species (ROS) production, and expression of genes related to antioxidant defense in yeast, such as the glutathione system, catalase, superoxide dismutase, glutaredoxin, and thioredoxin, were assessed. Melatonin alone decreased GSH, increased GSSG, and activated antioxidant defense system genes, which reached maximum levels in the stationary phase. These results indicate that melatonin supplementation enables cells to resist better the stress generated in the stationary phase. However, when cells were subjected to oxidative stress induced by H_2_O_2_, melatonin was able to partially mitigate cell damage by decreasing ROS accumulation and GSH and increasing GSSG; this was followed by enhanced cell viability after stress exposure, mostly when occurring in the early stationary phase. Additionally, under such conditions, most genes related to endogenous antioxidant defense continued to be up-regulated with melatonin supplementation. The findings demonstrate that melatonin can act as antioxidant in *S. cerevisiae*.

## Introduction

Melatonin (*N*-acetyl-5-methoxytryptamine) (MEL) is synthesized from tryptophan and exhibits various biological activities in humans. One such activity is its antioxidant capacity: MEL protects various biomolecules against damage caused by free radicals by acting as a direct scavenger to detoxify reactive oxygen and nitrogen species (Reiter et al., 2001, 2016; Anisimov et al., 2006). In addition, MEL can indirectly reduce oxidative stress by increasing the activities of antioxidative defense systems, stimulating the synthesis of other important intracellular antioxidants such as glutathione (Antolín et al., 1996; Rodriguez et al., 2004), increasing the efficiency of the mitochondrial electron transport chain (Martín et al., 2000; León et al., 2005; López et al., 2009) and interacting synergistically with other antioxidants (Gitto et al., 2001; López-Burillo et al., 2003). Most studies to date confirm the antioxidant properties of MEL, but it might also exert a pro-oxidant effect in specific situations, e.g., cancer cell killing, even though this is not well documented *in vivo* (Zhang and Zhang, 2014).

MEL can be found in small quantities in wines (74–420 ng/mL, Rodriguez-Naranjo et al., 2011), because it is present in grapes (Iriti et al., 2006; Murch et al., 2010; Stege et al., 2010) and is also synthesized by yeast during alcoholic fermentation (Rodriguez-Naranjo et al., 2012; Mas et al., 2014; Wang et al., 2016; Fernández-Cruz et al., 2017). Despite very little information is available on melatonin biosynthesis in yeast, its pathway is supposed to be similar to the one described in vertebrates, in which four enzymes are involved in the conversion of tryptophan to melatonin, via serotonin and *N*-acetylserotonin intermediates (Mas et al., 2014). However, the role of MEL in yeast remains unknown. *Saccharomyces cerevisiae* is the simplest eukaryote model and is also the main yeast used in the winemaking process, where is exposed to a number of stressors, each with the potential to cause cellular damage and impair fermentation performance (Pretorius, 2000; Gibson et al., 2007). One such stressor is oxidative stress, whereby yeast cells need to manage the toxic effects of reactive oxygen species (ROS) formed from molecular oxygen, including superoxide anion ($O_{2}^{•-}$), singlet oxygen (^1^O_2_), hydroxyl radical (OH^-^) or hydrogen peroxide (H_2_O_2_) (Moradas-Ferreira et al., 1996; Gibson et al., 2008). ROS are generated endogenously during normal cellular metabolism, and their production can also be stimulated by the presence of pro-oxidants. Under normal physiological conditions, yeast cells are able to maintain a reduced intracellular redox environment. However, ROS become harmful when their concentration exceeds the ability of the cells to remove them, causing respiratory deficiencies that result in oxidative stress. This oxidative stress can damage lipids, carbohydrates, proteins and nucleic acids, potentially leading to cell death (Moradas-Ferreira et al., 1996; Costa and Moradas-Ferreira, 2001; Gibson et al., 2008).

Yeast cells are constantly monitoring ROS concentrations in an attempt to maintain them at a basal level by invoking antioxidant defense mechanisms, which are grouped into enzymatic and non-enzymatic systems that operate at different levels (Jamieson, 1998; Moradas-Ferreira and Costa, 2000; Costa and Moradas-Ferreira, 2001). Enzymatic systems, which include catalase, superoxide dismutase, and glutathione peroxidase, are primary defenses that function to neutralize ROS. In contrast, non-enzymatic systems, such as the glutathione, glutaredoxin family or thioredoxins, are secondary defenses that repair or remove the products of oxidative damage (Jamieson, 1998). To eliminate ROS, cells need to be equipped with regulatory molecules that rapidly sense and respond to oxidative stress. In yeast, the parallel glutathione/glutaredoxin and thioredoxins pathways (essential under aerobic and anaerobic conditions) (Herrero et al., 2008) make a large contribution to protection against oxidative damage by reacting with ROS (Auchère et al., 2008). Glutathione (GSH) is well known as the main and most abundant endogenous antioxidant in cells (Izawa et al., 1995; Moradas-Ferreira et al., 1996; Jamieson, 1998). GSH reacts with ROS, donating an electron to neutralize them and becoming reactive itself, resulting in the formation of GSSG, the oxidized state, through the combination of two reactive forms of GSH. Thus, the presence of ROS results in a decrease in GSH and an increase in GSSG (Auchère et al., 2008; Herrero et al., 2008). The enzymes directly implicated in the maintenance of the GSH/GSSG redox balance are as follows: γ-glutamylcysteine synthetase, which catalyzes the first step in the biosynthesis of glutathione; glutathione reductase (GR), which reduces GSSG to GSH in an NADPH-dependent process; glutathione peroxidase, which reduces H_2_O_2_ by oxidizing GSH to GSSG; and glutaredoxins, which regulate the protein redox state using GSH and NADPH. Similar to glutaredoxins, thioredoxins are thiol oxidoreductases; however, in this case, they are not glutathione dependent but are only reduced by NADPH and thioredoxin reductase. Although cytosolic thioredoxin, encoded by *TRX2*, is the most important enzyme required for defense against externally added hydroperoxides, cytosolic glutaredoxin, encoded by *GRX2*, also contributes to resistance to hydroperoxides (Auchère et al., 2008; Herrero et al., 2008; Gómez-Pastor et al., 2012). Furthermore, *S. cerevisiae* possesses two catalases and two superoxide dismutases, which also act as enzymatic antioxidants. Catalase A and catalase T decompose H_2_O_2_ to oxygen and water in the peroxisome and the cytosol, respectively. Superoxide dismutases catalyze the conversion of superoxide anion to oxygen and H_2_O_2_ in the cytoplasm (Cu/ZnSOD) and in mitochondria (Mn/ZnSOD) (Jamieson, 1998; Costa and Moradas-Ferreira, 2001; Auchère et al., 2008). Most of these antioxidant mechanisms are considered universal in living organisms, and regulating expression of the genes involved as well as enzyme activity is crucial for cell survival.

The goal of this study was to evaluate the effect of MEL on *S. cerevisiae* and its possible role as an antioxidant. To accomplish this, we evaluated ROS production, intracellular glutathione levels (GSH/GSSG), and expression of certain genes involved in the oxidative stress response in a commercial wine yeast strain in both the presence and absence of MEL (5 μM) and oxidative stress (addition of 2 mM H_2_O_2_). Furthermore, as several studies have demonstrated that yeast cells in the stationary phase exhibit a significant degree of resistance toward oxidants (Jamieson, 1998; Gibson et al., 2008), the effect of MEL was evaluated in both the exponential and stationary phases.

## Materials and Methods

### Yeast Strain and Growth Conditions

The wine yeast QA23, a commercial strain of *S. cerevisiae* (Lallemand, Montreal, QC, Canada), was used in this study. For all experiments, after yeast rehydration, precultures for biomass propagation were prepared in YPD liquid medium [2% (w/v) glucose, 2% (w/v) peptone, and 1% (w/v) yeast extract] and incubated for 24 h at 28°C with orbital shaking (120 rpm).

### Determination of Reactive Oxygen Species (ROS)

A preliminary test to evaluate the concentration of H_2_O_2_ and MEL to be used in different experiments was carried out by determining their effect on the intracellular concentration of ROS.

The effect of H_2_O_2_ was first examined. Yeast cells were inoculated into 100 mL of YPD broth (5 × 10^5^ cells/mL) and grown for 6 h (until cells reached the exponential phase) at 28°C with orbital shaking at 120 rpm. The cells were then exposed to different concentrations of H_2_O_2_, (from 2 to 4 mM, Perdrogen^TM^, Sigma–Aldrich, St. Louis, MO, United States) for 1 h, and intracellular ROS were determined and compared to the control (without exposure to H_2_O_2_). Another assessment to fix MEL concentrations was performed. In this case, the same procedure was followed with the cells grown in the presence of different concentrations of MEL (0 μM, 5 μM, 25 μM or 50 μM) for 6 h.

Reactive oxygen species determination was carried out according to a modified version of the method described by Madeo et al. (1999) using dihydrorhodamine 123 (DHR 123; Sigma–Aldrich) as a ROS indicator. Cells were stained with 10 μg DHR 123 (stock solution of 2.5 mg/mL) per mL of cell culture for 15 min at 120 rpm in darkness. After incubation, the cells were washed twice with phosphate-buffered saline (PBS, pH 7.4), and the fluorescence intensity was analyzed by flow cytometry at a low flow rate with excitation and emission settings of 488 and 525–550 nm (filter FL1), respectively. FloMax software (Quantum Analysis GmbH, Münster, Germany) was used for instrument control and data acquisition, and the captured files were processed using WinMDI 2.9 software (Joseph Trotter, Salk Institute for Biological Studies, La Jolla, CA, United States). The mean fluorescence index (MFI) was calculated according to Boettiger et al. (2001): [(geometric mean of the positive fluorescence) – (geometric mean of the control)]/(geometric mean of the control). Moreover, cells were visualized using a Leica fluorescence microscope (DM4000B, Stuttgart, Germany) with a 40X lens.

### Experimental Conditions

Yeast cells were inoculated into 600 mL of YPD broth with and without supplementation of 5 μM MEL (MEL and Control, respectively) to obtain an initial population of 5 × 10^5^ cells/mL and grown for 24 h at 28°C with orbital shaking at 120 rpm. Both conditions were carried out in triplicate, and yeast growth was controlled by measuring the optical density at 600 nm (OD_600_) every 2 h, at which 1 × 10^8^ cells were transferred to 2-mL Eppendorf tubes and centrifuged. The pellets were washed twice with PBS (pH 7.4), frozen in liquid nitrogen and stored at -80°C for glutathione assays.

Sublethal oxidative stress was induced under both conditions by adding 2 mM hydrogen peroxide (H_2_O_2_) to the yeast cultures. In different phases of the growth curve [the early exponential phase (6 h), early stationary phase (16 h), and late stationary phase (30 h)], the Control and MEL conditions were divided into two flasks of 100 mL of culture each. Stress was induced in one flask of each condition with 2 mM H_2_O_2_ for 120 min to generate four conditions: Control and MEL (without stress); H_2_O_2_ and MEL H_2_O_2_ (with stress). Samples were collected before and after stress exposure (0, 10, 45, 90, and 120 min for glutathione quantification; 0, 45, and 120 min for gene expression) and stored as previously described. Three biological replicates were performed for each condition.

### Evaluation of Yeast Viability after Stress Exposure

Yeast viability after exposure to stress (MEL H_2_O_2_ and H_2_O_2_) in comparison with cells without stress (MEL and Control) was evaluated by a microplate bioassay in which 96-well plates were prepared by dispensing 250 μL of YPD broth inoculated with cells of each condition into each well to obtain an initial OD_600_ of 0.050. The microplate was incubated at 28°C for 24 h, and OD_600_ was measured every 30 min using a microplate reader (Omega Polarstar, BMG Labtech Gmbh, Ortenberg, Germany). OD max, growth rate and generation time were calculated from growth curves data, according to Warringer and Blomberg (2003). Moreover, the relative viable fraction was calculated using the formula described by Murakami et al. (2008):

$$Vn=\frac{1}{2^{\left(\frac{\Delta t_{n}}{\delta }\right)}}$$

where *Vn* = viability of the cultures exposed to stress (MEL H_2_O_2_ and H_2_O_2_) relative to cultures before stress exposure (MEL and Control, respectively), Δ*t*_n_ = time shift between the stressed and unstressed outgrowth curves to reach OD = 0.5, and δ = doubling time in each condition.

### Determination of Glutathione Levels

Samples (1 × 10^8^ cells) were rapidly thawed in a water bath at 37°C. For glutathione extraction, a modified version of the method described by Borrull et al. (2016) was used. Pellets (previously dried at 28°C for 48 h) were weighed, and three volumes of 5% 5-sulfosalicylic acid (SSA) were added and vortexed. The cell suspensions were then frozen in liquid nitrogen and thawed at 37°C in a water bath three times, incubated for 5 min at 4°C and centrifuged at 800 × *g* for 10 min at 4°C. For quantification, GSSG first needs to be reduced to GSH. This enzymatic reduction was performed using GR (Sigma–Aldrich) and the cofactor NADPH (Sigma–Aldrich). Briefly, 50 μL of homogenate and three units of GR solution were dissolved in 950 μL PBS (pH 7.8) with 16 mg/mL NADPH and incubated at 25°C for 10 min. Total glutathione (GSHtot) and GSH were determined using the method described by White et al. (2003). Briefly, 20 μL of supernatant was transferred to a 96-well plate designed for fluorescence detection, and 180 μL of 2,3-naphthalenedicarboxyaldehyde (NDA) derivatization solution (50 mM Tris, pH 10, 0.5 N NaOH, and 10 mM NDA in Me_2_SO, v/v/v; Sigma–Aldrich) was added. The microplate was shaken for 10 min at 150 rpm and at 20 ± 2°C in darkness, as recommended by Lewicki et al. (2006), to maintain stability of the NDA-GSH adduct. After this incubation, fluorescence intensity was measured (488 ex/530 em) using a fluorescence plate reader. For total and reduced glutathione quantification, linear regression curves were generated using GSSG and GSH standard solutions (Sigma–Aldrich). The concentration of GSSG was calculated by subtracting reduced GSH from total GSH and dividing this value by 2. The results are expressed as μM of glutathione per mg of dry weight.

### Gene Expression Analysis by Quantitative PCR (qPCR)

Expression levels of specific genes (**Table 1**) were determined using qPCR. Total RNA from 1 × 10^7^ cells was isolated using a PureLink^®^ RNA Mini kit from Ambion Life Technologies (Woburn, MA, United States) as recommended by the manufacturer. To remove DNA, a DNAse (Qiagen, Barcelona, Spain) step was performed at 37°C for 15 min before washing. Reverse transcription and qPCR reactions were performed as described by Beltran et al. (2004). cDNA was synthesized from 320 ng/μL RNA using SuperScript^®^ III Reverse Transcriptase (Invitrogen) and Oligo (dT) 20 Primer (Invitrogen). qPCR was performed using the Applied Biosystems 7300 Fast Real-Time PCR system (Applied Biosystems, Foster City, CA, United States). Samples were prepared as follows: 2 μL cDNA, 0.4 μL each primer, 0.4 μL ROX, 10 μL SYBR Green (Takara^®^ SYBR Green master mix), and H_2_O q.s.p. 20 μL. Relative gene expression was calculated using the 2^-ΔΔCt^ formula, where Ct is defined as the cycle at which fluorescence is determined to be statically significantly above background; ΔCt is the difference in Ct of the gene of interest and the housekeeping gene (*ACT1*), and ΔΔCt is the difference between ΔCt of the condition MEL at 6 h or 16 h and the Control at 6 h or 16 h (see figure legends for relative expression details). Three biological replicates were analyzed for each time point and condition.

**Table 1:** Primers used in this study based on, Auchère et al. (2008), Verbelen et al. (2009), and Gómez-Pastor et al. (2012) (supplied by Invitrogen).

Gene description	Primer	Nucleotide sequence (5′ to 3′)
Cu/Zn superoxide dismutase	*SOD1_F*	TGATCAAGCTTATCGGTCCTACCT
	*SOD1_R*	GCCGGCGTGGATAACG
Mn superoxide dismutase	*SOD2_F*	GCAAGCTGGACGTTGTTCAA
	*SOD2_R*	AGAGGAACTAGTGGGCCTGTGA
Peroxisomic catalase A	*CTA1_F*	GGACAGCAAAAGAACTTGGCATA
	*CTA1_R*	TGAGGACAGGCGCCTTCTA
Cytosolic catalase T	*CTT1_F*	GTCAGGCTCCCACCCTGAT
	*CTT1_R*	TTTTCGCCATTTTGCAATTG
Glutathione peroxidase I	*GPX1_F*	GGGAAGTCTGGAATAAAAATGATAAA
	*GPX1_R*	TTCTTCTGGTGGTTGATTCAGTA
γ-Glutamylcysteine synthetase	*GSH1_F*	GACACCGATGTGGAAACTGA
	*GSH1_R*	CCCTTTTTGGCATAGGATTG
Glutathione-disulfide reductase	*GLR1_F*	AGGTTGTCGGTCTGCACATT
	*GLR1_R*	CCTTAGTGGCACCCATCTTT
Glucose-6-phosphate dehydrogenase	*ZWF1_F*	CCAGAGGCTTACGAGGTGTT
	*ZWF1_R*	GGTGAATATGCCCCAACTGA
Glutaredoxin	*GRX2_F*	GGCCAAAAGGAAGTGTTTGT
	*GRX2_R*	TTCAATTCTTGGAAGAGGGTAGA
Thioredoxin	*TRX2_F*	AAATCCGCTTCTGAATAC
	*TRX2_R*	CTATACGTTGGAAGCAATAG


### Data Analysis

Data were subjected to one-way analysis of variance (ANOVA) and Tukey’s *post hoc* test to evaluate the effect of each treatment. The results were considered statistically significant at a *p-*value less than 0.05 (IBM SPSS Inc, XLSTAT Software). Furthermore, a Principal Component Analysis (PCA) was performed at 6, 16, and 30 h (XLSTAT Software). PCs were assessed using glutathione levels (GSH and GSSG, at 10, 45, 90, and 120 min), and growth data (OD max, and maximum growth rate calculated at 5, 10, 15, and 20 h).

## Results

### Effect of Melatonin on Reactive Oxygen Species (ROS)

**FIGURE 1:**
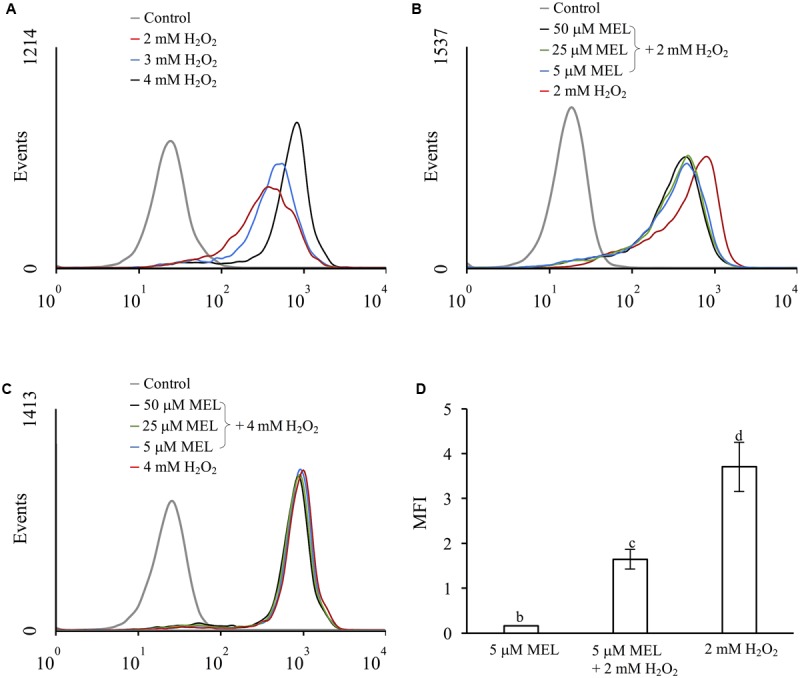
Effect of H_2_O_2_ and melatonin on ROS accumulation, evaluated in the exponential phase. Flow cytometry histogram profile expressed in number of events. **(A)** 0, 2, 3 and 4 mM H_2_O_2_ in the absence of melatonin; **(B)** 2 mM H_2_O_2_ and **(C)** 4 mM H_2_O_2_ in presence of melatonin (5, 25 and 50 μM). The control condition corresponds to unstressed cells without melatonin. **(D)** Mean fluorescence intensity (MFI) of unstressed and stressed cells (2 mM H_2_O_2_) in the presence of melatonin (5 μM), compared with the control condition. Error bars represent SD of *n* = 3 by ANOVA and Tukey’s post-test (b,c,d), *p* < 0.05.

To evaluate the possible role of MEL as an antioxidant agent in *S. cerevisiae*, we determined the levels of ROS in stressed and unstressed cells (using H_2_O_2_ as the oxidative agent) in the presence and absence of MEL in the growth medium (**Figure 1**). Although *S. cerevisiae* can synthetize MEL, we have previously observed that in these conditions, the concentration of MEL in the extracellular medium is negligible (below 0.4 nM, data not shown). Preliminary experiments were conducted to select a sublethal dose of H_2_O_2_ and the concentration of MEL with a possible antioxidant effect. Exposure to increasing concentrations of H_2_O_2_ resulted in an increase in ROS (**Figure 1A**). However, when the oxidative stress was applied to cultures growing with MEL (from 5 to 50 μM), a reduction in ROS was only observed at 2 mM H_2_O_2_ (**Figure 1B**); this reduction was dependent on MEL addition but independent of the MEL concentration in the medium. In contrast, no effect on ROS accumulation was observed when cells were exposed to higher H_2_O_2_ concentrations (4 mM, **Figure 1C**). Thus, we chose the lowest assayed dose of MEL (5 μM) and H_2_O_2_ (2 mM) for the ensuing experiments. As shown in **Figure 1D**, cells exposed to oxidative stress (2 mM H_2_O_2_) exhibited strong increases in total ROS, with four times higher levels than unstressed cells (**Figure 1D**). However, ROS accumulation in cells under the same oxidative stress conditions but previously grown in presence of MEL was significantly lower (only two times higher than unstressed cells). Conversely, a low dose (5 μM) of MEL alone, without the presence of the oxidative agent, resulted in slightly increased total ROS.

### Effect of Melatonin on the Glutathione Redox Status of the *S. cerevisiae* Wine Strain

**FIGURE 2:**
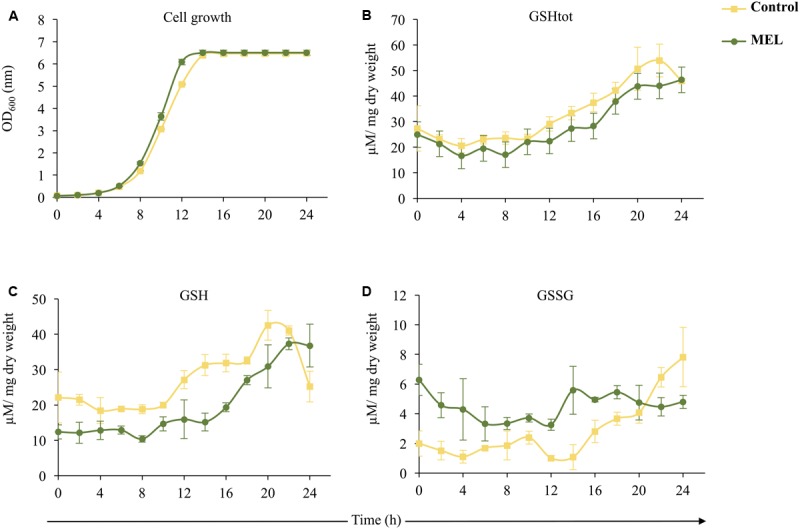
Effect of the presence (MEL; 5 μM) or absence (Control) of melatonin on intracellular glutathione levels in *Saccharomyces cerevisiae* (QA23 strain) over 24 h of growth in YPD. Error bars represent ± SD of *n* = 3. **(A)** Growth of the QA23 strain (OD, optical density). **(B)** Total glutathione (GSHtot) levels. **(C)** Reduced glutathione (GSH) levels. **(D)** Oxidized glutathione (GSSG) levels.

To further study the role of MEL in yeast cells, we evaluated the effect of its supplementation (5 μM MEL) on intracellular glutathione levels by analyzing both reduced (GSH) and oxidized (GSSG) glutathione over 24 h of growth (**Figure 2**). Although similar growth curves were observed for both conditions (with and without MEL), with MEL, cells grew faster during the exponential phase (**Figure 2A**). Total GSH remained almost constant until the mid-exponential phase, when it began to increase, with a similar pattern observed in both conditions; however, GSHtot levels were slightly lower in the presence of MEL (**Figure 2B**). Despite the lack of significant changes in GSHtot when MEL was added, the glutathione ratio (GSH/GSSG) with and without MEL supplementation differed. In the presence of MEL, cells exhibited low levels of GSH and high levels of GSSG during the first 20–22 h (**Figures 2C,D**). GSH evolution showed similar trends with and without MEL until 22 h. As for GSHtot, the concentration of GSH was almost constant until the mid-log phase and then increased to reach its maximum value at 20–22 h. After this point (22 h), the level of GSH decreased without MEL but remained constant with MEL (**Figure 2C**). Although the GSSG content was higher in the culture supplemented with MEL, its concentration remained essentially unchanged during the 24 h of study. Without MEL, the GSSG concentration increased after the mid-log phase and was higher under this condition at 22–24 h than in the presence of MEL (**Figure 2D**).

### Effect of Melatonin on Expression Levels of Genes Related to the Antioxidant Response

**FIGURE 3:**
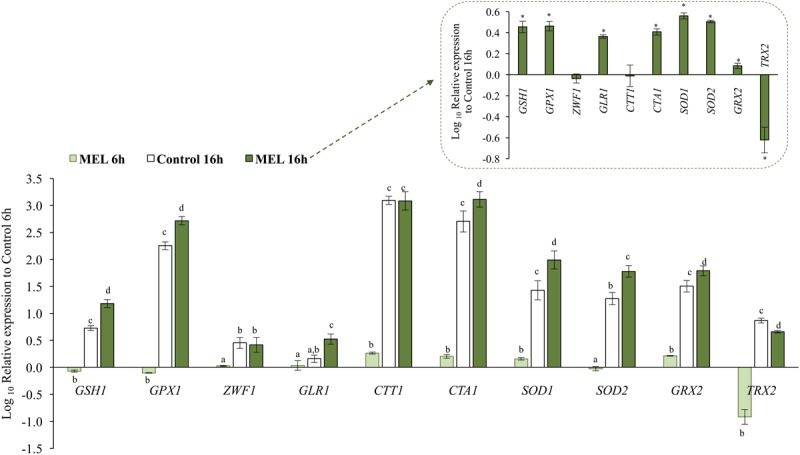
Effect of melatonin on expression of genes encoding enzymes involved in the response to oxidative stress: γ-glutamylcysteine synthetase (*GSH1*), glutathione peroxidase (*GPX1*), glucose 6-phosphate dehydrogenase (*ZWF1*), glutathione reductase (*GLR1*), catalase T and A (*CTT1* and *CTA1*, respectively), Cu/ZnSOD (*SOD1*), MnSOD (*SOD2*), glutaredoxin (*GRX2*), and thioredoxin (*TRX2*). Gene expression was determined at the early exponential (6 h) and early stationary (16 h) phases with and without melatonin (MEL and Control, respectively). The values are expressed relative to expression at 6 h without melatonin (Control 6 h). Relative expression of all genes in MEL 16 h relative to Control 16 h are also shown in the inset. Error bars represent ± SD of *n* = 3 by ANOVA and Tukey’s post-test (a–d) or ^∗^ *p* < 0.05.

Quantitative PCR was used for transcriptional analysis of certain genes implicated in endogenous antioxidant defense, such as *CTA1* and *CTT1* (catalase A and T, respectively), *SOD1* and *SOD2* (cytoplasmic and mitochondrial superoxide dismutase, respectively), *GRX2* (glutaredoxin), and *TRX2* (thioredoxin) and in glutathione metabolism, such as *GSH1* (γ-glutamylcysteine synthetase), *GLR1* (GR), *GPX1* (glutathione peroxidase), and *ZWF1* (glucose-6-phosphate dehydrogenase, which reduces NADP^+^ to NADPH). The effect of MEL supplementation (5 μM) on the expression levels of all these genes was determined in the early exponential (6 h) and early stationary (16 h) phases (**Figure 3**). After 6 h in the presence of MEL, *CTT1*, *CTA1, SOD1*, and *GRX2* expression was higher than that under the control condition, whereas expression of *GSH1*, *GPX1*, and especially *TRX2*, was lower. Conversely, expression of *GLR1, ZWF1*, and *SOD2* was not affected.

Upon entry into the stationary phase (16 h), expression of all genes increased significantly under the control condition (**Figure 3**), with the highest levels found for catalase genes (*CTT1* and *CTA1*; 1000 and 600 times, respectively) and the lowest for *GLR1* and *ZWF1* (2 and 3 times, respectively). Moreover, at this time point, the presence of MEL resulted in a greater increase in the expression level of most genes (*GSH1*, *GPX1*, *GLR1*, *CTA1*, *SOD1*, *SOD2*, and *GRX2)*, between 3 and 5 times higher than under the control condition (**Figure 3**, inset). Exceptions were *CTT1* and *ZWF1*, expression of which was similar to the control, and *TRX2*, expression of which remained lower than the control.

MEL activated the cytosolic catalase gene (*CTT1*) but only in the early exponential phase. Instead, at the stationary phase (16 h) expression of *CTT1* was highly up-regulated, regardless of the presence of MEL (**Figure 3**).

### Effect of Melatonin on the Glutathione Redox Status under Oxidative Stress

**FIGURE 4:**
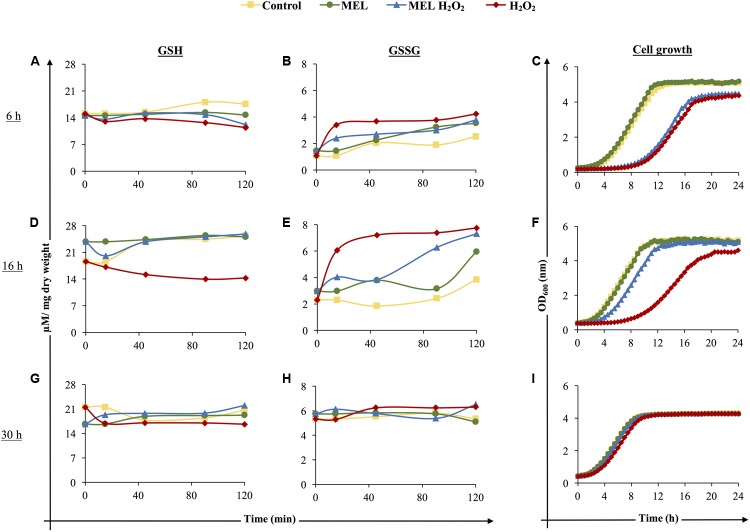
Effect of the presence of melatonin on reduced glutathione (GSH: **A,D,G**), oxidized glutathione (GSSG: **B,E,H**), and viability **(C,F,I)** of unstressed and stressed cells before and at 10, 45, 90, and 120 min after oxidative stress induction. Control: control cells; MEL: 5 μM melatonin; MEL H_2_O_2_: 5 μM melatonin and 2 mM H_2_O_2_; H_2_O_2_: 2 mM H_2_O_2_. Stress was applied in the early exponential phase (6 h, **A,B,C**), in the early stationary phase (16 h, **D,E,F**), and in the late stationary phase (30 h, **G,H,I**). For the viability test, cells from the four conditions were reinoculated in YPD fresh medium.

To evaluate the effect of MEL on the glutathione redox balance in yeast under oxidative stress, the response to the addition of an oxidant compound such as H_2_O_2_ in the presence or absence of MEL was assessed at different phases of growth. Glutathione levels (GSH and GSSG) were analyzed before and after stress induction with 2 mM H_2_O_2_ (at 0, 10, 45, 90, and 120 min of exposure) and at different stages of growth [early exponential (6 h), early stationary (16 h), and late stationary (30 h) phases]. To determine the ability of cells to grow after stress exposure, cells were reinoculated in YPD, and growth was followed for 24 h. The levels of GSH/GSSG and cell growth recovery differed depending on the time at which the stress was applied (**Figures 4A–I**). The presence of MEL at the early exponential phase, as shown in **Figure 2**, caused small differences in intracellular glutathione levels, slightly decreasing GSH and increasing GSSG (**Figures 4A,B**), and these changes did not alter cell growth recovery (**Figure 4C**). When oxidative stress was applied (H_2_O_2_), the redox balance changed, and a significant increase in GSSG/decrease in GSH was detected. Moreover, the relative viable fraction, calculated from cell growth curves, dramatically decreased until 43.0 ± 1.2% (considering 100% the value of Control condition), indicating that the initial viability was highly affected by stress. In consequence, cell growth was highly affected, presenting a longer lag phase, higher generation time and lower final cell concentration (**Figure 4C**). This damage was slightly mitigated when stress was applied in the presence of MEL (MEL H_2_O_2_), with lower GSSG accumulation and slightly higher GSH than in stressed cells in the absence of MEL. Although the viable fraction was also affected, this value was higher with MEL supplementation (51.9 ± 1.8%), resulting in a slight improvement of cell growth (**Figure 4C**).

At the early stationary phase, the total amount of glutathione (GSH and GSSG) was higher under all conditions (**Figures 4D,E**), as shown in **Figure 2B**. When stress was applied, larger differences between stressed and non-stressed cells were observed, with again lower levels of GSH and higher levels of GSSG in stressed cells. Moreover, recovery of cell growth and viability was also strongly affected by stress exposure (**Figure 4F**), presenting a relative viable fraction of 60.0 ± 1.5%. Instead, the presence of MEL under oxidative stress significantly decreased GSSG and increased GSH, reaching similar levels as non-stressed cells (**Figures 4D,E**). In addition, the relative viable fraction in presence of MEL was significantly higher (80.9 ± 2.9%), greatly enhancing cell growth after stress, with a growth curve similar to non-stressed cells (**Figure 4F**).

Finally, when stress was applied at 30 h, GSH/GSSG levels were similar for all conditions, with the only significant differences found at 120 min after stress exposure, i.e., lower GSH (**Figure 4G**) and higher GSSG (**Figure 4H**) levels. In this case, the decrease of the relative viable fraction was similar within both stressed conditions (MEL H_2_O_2_: 94.8 ± 1.9%; H_2_O_2_: 91.8 ± 1.5%) and much higher than in early exponential and stationary phases, allowing the cells to normally recover growth after stress exposure (**Figure 4I**). The presence of MEL did not significantly modify the glutathione profile or the growth curve at this stage. In fact, under all conditions (Control, MEL, MEL H_2_O_2_ and H_2_O_2_), similar population sizes were achieved at the stationary phase, which were lower than those after oxidative stress exposure at 6 h or 16 h (**Figure 4I**).

**FIGURE 5:**
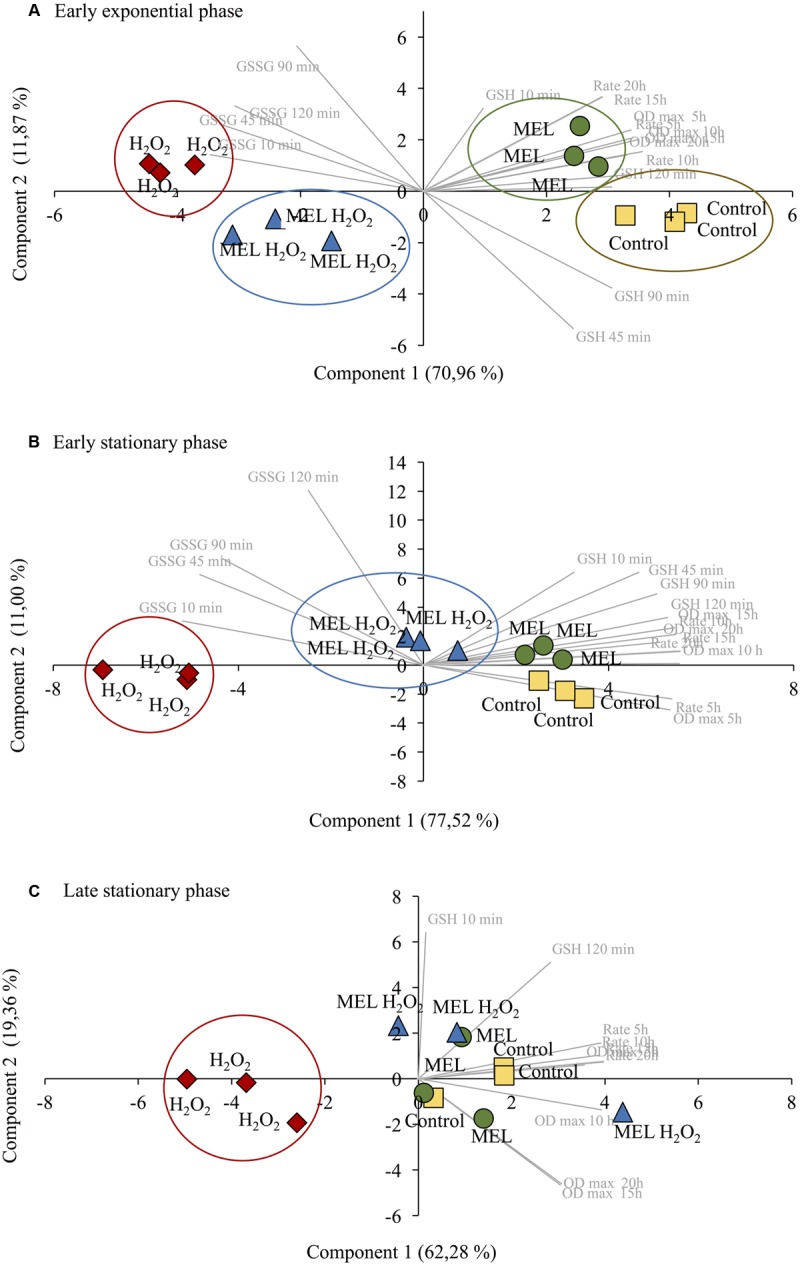
Bi-plots of principal components analysis (PCA) using the following variables: intracellular reduced GSH, oxidized GSH (GSSG), maximum optical density (OD max), and maximum growth rate values calculated at 5, 10, 15, and 20 h based on cell growth obtained in the viability test. Control: control cells; MEL: 5 μM melatonin; MEL H_2_O_2_: 5 μM melatonin and 2 mM H_2_O_2_; H_2_O_2_: 2 mM H_2_O_2_. **(A)** Oxidative stress applied at early exponential phase (6 h). Component 1: (+); GSH (10, 45, 90, and 120 min), OD max and growth rate (5, 10, 15, and 20 h). (–); GSSG (10, 45, and 120 min). Component 2: (+); GSSG 90 min. **(B)** Oxidative stress applied at early stationary phase (16 h). Component 1: (+); GSH (10, 45, 90, and 120 min), OD max and rate (5, 10, 15, and 20 h). (–); GSSG (0, 45, and 90 min). Component 2: (+); GSSG 120 min. **(C)** Oxidative stress applied at late stationary phase (30 h). Component 1: (+); GSH 120 min, OD max (5, 10, 15, and 20 h), growth rate (5 and 10 h). Component 2: (+); GSH 10 min.

For further analysis of these data (**Figure 4**), a PCA was applied to correlate the different variables (reduced and oxidized glutathione and growth curves data) and highlight if there were grouping patterns within the different conditions (Control, MEL, MEL H_2_O_2_, H_2_O_2_) at 6, 16, and 30 h (**Figure 5**). In the resulting PCA plot at 6 h (**Figure 5A**) and 16 h (**Figure 5B**), the PCs explained 82.83 and 88.52% of the variance, respectively. In both PCA, parameters indicating greater viability (higher rate and OD max) were positively correlated with higher levels of GSH (positive component 1) and negatively correlated with higher levels of GSSG (negative component 1) (**Figures 5A,B**). Thus, when stress was applied in the early exponential phase (6 h, **Figure 5A**), the different conditions were clearly separated into four groups, where cells without stress presented better growth, higher GSH and lower GSSG levels than stressed cells. MEL condition was grouped apart from the control due to a higher oxidized state (higher GSSG and lower GSSG). In contrast, in MEL H_2_O_2_, a decrease in the oxidized state in comparison to stressed cells resulted in a shift in the component 1 toward the unstressed conditions, being this shift even greater when the stress was applied at 16 h (**Figure 5B**). Finally, in the late stationary phase (30 h), the PCs explained 81.64% of the variance (**Figure 5C**), but merely by the cellular growth variables and GSH at 120 min (positively correlated within the positive component 1). Only stressed cells without MEL were grouped together and separated from the other conditions, presenting the lowest GSH levels at 120 min, rate growth and OD max, what indicated that MEL also has a slight effect when the stress was applied at 30 h.

### Effect of Melatonin on Expression Levels of Genes Related to the Antioxidant Response under Oxidative Stress

**FIGURE 6:**
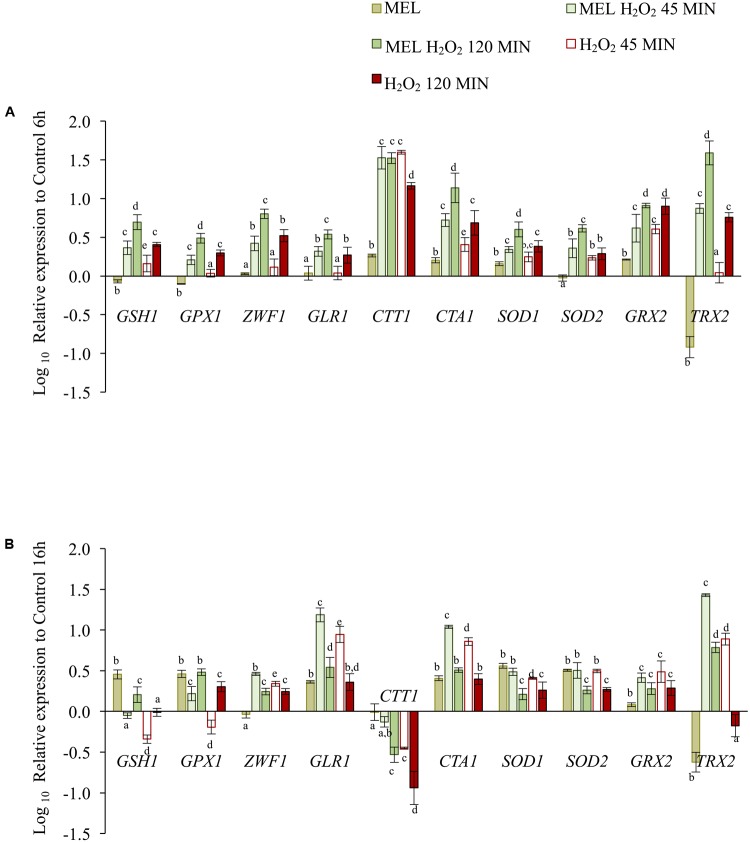
The effect of melatonin on expression of genes involved in the oxidative stress response, as determined before and after 45 and 120 min after stress exposure (2 mM H_2_O_2_), in cells previously grown with MEL (5 μM) and without MEL. **(A)** Gene expression in the early exponential phase, when stress was applied at 6 h. **(B)** Gene expression in the early stationary phase, when stress was applied at 16 h. Changes are expressed relative to gene expression of the Control condition at time 6 h or 16 h. Error bars represent ± SD of *n* = 3 by ANOVA and Tukey’s post-test (a–d), *p* < 0.05.

Expression of selected genes implicated in endogenous antioxidant defense was also determined at 45 and 120 min after oxidative stress (2 mM H_2_O_2_) applied in the early exponential (6 h) or early stationary (16 h) phase and in the presence or absence of MEL (**Figure 6**).

In the early exponential phase (**Figure 6A**), the expression levels of all genes increased significantly at 120 min after stress exposure, with six of increasing already at 45 min (*GSH1*, *CTT1*, *CTA1*, *SOD1*, *SOD2*, and *GRX2*). The presence of MEL in stressed cells resulted in faster up-regulation of most genes (except for *CTT1* and *GRX2*), as the levels obtained at 45 min with MEL (MEL H_2_O_2_) were similar to those obtained at 120 min without MEL (H_2_O_2_). Indeed, at 120 min, the expression levels of all genes (except *GRX2*) were significantly higher with MEL than without. Exposure to stress at 6 h caused a 30-fold increase in expression of the *CTT1* gene at 45 min, an activation that was much lower than that observed upon entry into the stationary phase (**Figure 3**). However, at 120 min, expression remained constant in the presence of MEL but declined in its absence. In general, the levels of gene up-regulation obtained by exposure to stress during the log phase (6 h) were lower than those obtained upon entry into the stationary phase (**Figure 3**), except for *TRX2*, the levels of which were higher under stress in the presence of MEL.

The observed expression profile was different in cells stressed in the stationary phase (16 h) (**Figure 6B**). In this case, most of the genes (*ZWF1*, *GLR1*, *CTA1*, *SOD1*, *SOD2*, *GRX2*, and *TRX2*) were quickly activated after stress exposure, with higher values at 45 min, though their expression levels decreased over time. The presence of MEL increased expression of some of these genes (*ZWF1*, *GLR1*, *CTA1, SOD1*, and *TRX2*) but only during the first 45 min. At 120 min, expression of only *GSH1*, *GPX1*, and *TRX2* remained high in the presence of MEL (MEL H_2_O_2_). Compared to non-stressed cells, *CTT1* expression at the stationary phase was lower in stressed cells, with the lowest levels in the absence of MEL. *GSH1* and *GPX1* also exhibited lower levels of expression than the control condition at 45 min after stress exposure, though these levels were up-regulated over time, with higher levels of expression in the presence of MEL.

As mentioned above, MEL alone was able to up-regulate certain genes in cells not exposed to stress, yet this increase in expression due to the presence of MEL at 6 h was much lower than the levels observed after stress exposure. For most genes (*GSH1*, *GPX1*, *GLR1*, *CTA1*, *SOD1*, and *SOD2*), this increase at 16 h was similar or even higher than that obtained after 120 min of stress exposure.

## Discussion

Recently, it has been described that *S. cerevisiae* synthetizes bioactive compounds derived from aromatic amino acids such as MEL during alcoholic fermentation. The role of MEL in cells has been extensively studied in humans and other organisms (Hardeland and Poeggeler, 2003; Tan et al., 2015), and its antioxidant capacity is among the most important biological activities described. However, its role in yeast is unknown. Therefore, in this study, the possible effect of MEL in protecting against oxidative stress was evaluated in *S. cerevisiae*, a well-established eukaryotic model and considered the wine yeast par excellence.

Exposure to oxidative stress generates ROS, which adversely affect cells when their capacity to eliminate these reactive species is exceeded. Therefore, cells need to be equipped with regulatory molecules to rapidly sense and respond to oxidative stress. We have focused our research on GSH as the main and most abundant endogenous antioxidant in cells (Moradas-Ferreira et al., 1996; Jamieson, 1998), and accordingly, the effect of MEL on the glutathione status with and without stress was evaluated in *S. cerevisiae* in the current study. Our results show that the presence of MEL at low doses (5 μM) alters basal glutathione levels with a slight increase in ROS accumulation. ROS accumulation with a decrease in GSH due to MEL has been exclusively reported in the *in vitro* response in human cells, mostly in cancer cells in which MEL exhibits pro-oxidant properties (Osseni et al., 2000; Wölfler et al., 2001; Albertini et al., 2006). Interaction between calmodulin and MEL might represent the mechanism involved in the stimulation of ROS by MEL (Radogna et al., 2009). We also observed that MEL repressed the *GSH1* and *GPX1* genes, which encode γ-glutamylcysteine synthetase and glutathione peroxidase, respectively, in the early exponential phase. Such repression by the presence of MEL has not previously been reported. These results confirm that the reduced ratio of GSH:GSSG corresponds to a decrease in mRNA levels of γ-glutamylcysteine synthetase. Many studies have documented the effects of MEL on gene regulation in human cells, and the mechanism by which MEL alters expression of antioxidant genes in *S. cerevisiae* may also be mediated by MEL receptor activation (Rodriguez et al., 2004; Tomás-Zapico and Coto-Montes, 2005). In this way, MEL would act indirectly on the glutathione system by decreasing GSH. Furthermore, thioredoxin, encoded by *TRX2*, which is specialized in protection against ROS, was strongly repressed in the presence of MEL, even though its mRNA levels were derepressed over time. This fact could explain the observed slight accumulation of ROS. Furthermore, very low doses of ROS (non-toxic levels) can serve as signaling molecules for cells to adapt and become more resistant to a subsequent lethal exposure.

Simultaneously, MEL activates genes involved in primary defense, which are normally repressed or present at very low levels during anaerobic growth in high-glucose culture media (Jamieson, 1998; Belazzi et al., 1991; DeRisi et al., 1997; Boy-Marcotte et al., 1998; Puig and Pérez-Ortín, 2000; Büyükavci et al., 2006), including those encoding both catalases (*CTT1* and *CTA1*) and cytosolic superoxide dismutase (*SOD1*). However, in the early stationary phase, when glucose was consumed by the cells (data not shown), all genes examined in the study were derepressed, and their expression increased. The transition of *S. cerevisiae* from fermentative to respiratory metabolism increases ROS production and involves modulation of the antioxidant system that confers cell resistance to oxidants. For this reason, as our results confirmed, cells are more susceptible to stress during the exponential phase than during the stationary phase. This also agrees with the viability results of cells after stress exposure, where the viability increased when the stress was applied in early and late stationary phase. In addition to activation of these genes in the early stationary phase, a clear effect of MEL was observed because it potentiated expression of many genes involved in primary (*GPX1*, *CTA1*, *SOD1*, and *SOD2*) and secondary (*GSH1*, *GLR1*, and *GRX2*) defense systems. ROS accumulation and changes in the basal glutathione balance followed by activation of stress genes in the presence of MEL had no effect on *S. cerevisiae* viability, indicating a lack of correlation with cytotoxicity or apoptosis (Osseni et al., 2000; Büyükavci et al., 2006; Girish et al., 2013). Our results appear to indicate that MEL prepared the cells to better endure stress generated in the stationary phase by inducing an increase in mRNA levels of antioxidant genes.

Nonetheless, exposure to oxidative stress (2 mM of H_2_O_2_) caused an increase in ROS, which activated defense mechanisms to maintain a proper redox state. Upon H_2_O_2_ challenge, yeast cells activate various antioxidant functions, including a gene expression program mediated largely by the transcription factors Msn2p/4p in a general stress response and Yap1p and Skn7p in a specific response to oxidative stress. (Moradas-Ferreira et al., 1996; Jamieson, 1998; Moradas-Ferreira and Costa, 2000; Costa and Moradas-Ferreira, 2001). Skn7p factor controls a subset of genes involved in the thioredoxin system, whereas Yap1p is required for the induction of all the oxidative responsive genes (Gómez-Pastor et al., 2010). The effect of stress on *S. cerevisiae* cells was higher in the early exponential (6 h) and stationary (16 h) phases than in the late exponential phase (30 h). At 30 h, the cells appeared to have already prepared their defense mechanisms and be more resistant to oxidants. Under such stress conditions, our results clearly showed, as have the vast majority of studies in humans (Tan et al., 2015; Reiter et al., 2016), that MEL has an antioxidant effect on *S. cerevisiae* in the early exponential and early stationary phases. Although MEL mitigated ROS accumulation generated by low concentrations of H_2_O_2_ in *S. cerevisiae*, this effect was not observed at higher concentrations of H_2_O_2_ (4 mM), likely because the oxidative stress exceeded the endogenous antioxidant capacity. Additionally, MEL-treated cells exhibited increased GSH and decreased GSSG concentrations. This state of lower oxidative stress resulted in a clear enhancement in cell viability in the early stationary phase. In humans, MEL displays multiple mechanisms to protect cells against oxidative stress, e.g., it possesses direct free radical scavenging activity, whereby it is able to detoxify ⋅OH produced by H_2_O_2_ (Tan et al., 2000). In our case, analysis of expression of certain stress-related genes under oxidative stress appeared to indicate that MEL in *S. cerevisiae*, as in humans, might interact with different components of antioxidant defense systems. In fact, the amphiphilic characteristics of MEL allow it to cross all morpho-physiological barriers, reaching any subcellular structure and guaranteeing its ability to act as a free radical scavenger (Tan et al., 2000; Reiter et al., 2007). Although MEL is able to act as a direct radical scavenger in *S. cerevisiae*, our results showed that MEL might also function as an indirect antioxidant by increasing the transcription level of genes related to the antioxidant response. Furthermore, stimulation of expression of genes encoding antioxidant enzymes by MEL via receptor activation can occur at nanomolar concentrations in cultured cells (Kotler et al., 1998; Mayo et al., 2002; Rodriguez et al., 2004).

As discussed above, an intense change in gene activity occurred within minutes after exposure to H_2_O_2_ in both the early exponential and stationary phases, and our results evidenced how low concentrations of MEL influenced even more changes in gene expression. A number of *in vitro, in vivo*, and *ex vivo* studies in humans have documented the ability of MEL to increase expression of multiple antioxidant stress genes, including copper zinc and manganese superoxide dismutases, glutathione peroxidase, catalase, and γ-glutamylcysteine synthase (Kotler et al., 1998; Esparza et al., 2005; Gómez et al., 2005; Mauriz et al., 2007; Fischer et al., 2013). Moreover, this effect on antioxidant enzyme genes appears to be consistent with the ability of MEL to up-regulate the activities of these enzymes as well as GR and glucose-6-phosphate dehydrogenase. In the current study, as in mammalian studies, the presence of MEL induced high levels of *GSH1*, *GPX1*, *CTT1*, *CTA1*, *SOD1*, and *SOD2* mRNA (Mayo et al., 2002; Tomás-Zapico and Coto-Montes, 2005; Kotler et al., 1998). Furthermore, our results showed that in *S. cerevisiae*, MEL also up-regulated expression of *GLR1*, *ZWF1* and particularly *TRX2*, which encodes the most important hydrogen peroxide-eliminating enzyme. Conversely, *CTT1* expression exhibited a different profile: it was rapidly activated by H_2_O_2_ in the early exponential phase, but it was repressed in the early stationary phase, showing an earlier decrease in gene expression in the absence of MEL. *CTT1*, which encodes cytosolic catalase T, is involved in the primary antioxidant defense induced by H_2_O_2_. This gene is not only induced by hyperoxidant conditions but also by starvation and heat or osmotic shock (Moradas-Ferreira et al., 1996; Jamieson, 1998; Costa and Moradas-Ferreira, 2001). Our results showed that induction of this gene occurred mainly during the exponential phase and that the effect of MEL on *CTT1* was significantly reduced after this phase. Nonetheless, some effect was still observed because lower levels were reached in the absence of MEL. *ZWF1* stimulation by MEL has been suggested in a few studies, as activation of the enzyme glucose-6-phosphate dehydrogenase was observed in the presence of the molecule (Pierrefiche and Laborit, 1995; Hardeland, 2005). The overexpression of *ZWF1* due to MEL under stress conditions observed in our study appears to highlight the importance of this gene in GSH recycling, as NADPH is a necessary cofactor for GR and provides reducing equivalents for redoxin systems. *TRX2*-encoded thioredoxin, the mRNA levels of which were up-regulated with MEL addition under oxidative stress conditions, is specialized in protecting against ROS and is essential for YAP1-dependent resistance to hydroperoxides (Kuge and Jones, 1994; Herrero et al., 2008; Gómez-Pastor et al., 2012); indeed, this gene (*TRX2*) is one of the first targets of the major oxidative stress transcription factor Yap1p. *TRX2*, besides to be required to regulate redox state and levels of glutathione in response to oxidants, it can be also up-regulated in response to changing growth conditions, providing a first line of defense against oxidative stress (Gómez-Pastor et al., 2012). Therefore, our results indicate that MEL indirectly increases the resistance of *S. cerevisiae* to oxidative stress via overexpression of *TRX2*, enhancing the fermentative capacity and viability of this wine strain under vinification conditions.

MEL has frequently been compared with vitamins C and E in terms of antioxidant properties (Reiter et al., 2007). The effect of MEL on *S. cerevisiae* may also be comparable to the effect of resveratrol on yeast (Escoté et al., 2012), which induces Yap1p activity (the major regulator of genes involved in the oxidative stress response) to reduce ROS levels under oxidative stress.

The literature contains many references regarding the biological properties of MEL as a hormone and its antioxidant properties, among other beneficial effects in vertebrates. Based on our results, MEL synthesis by *S. cerevisiae* during wine production could be related to the ability of yeast cells to adapt to and endure the hostile environment of the wine-making process and probably counteract the pro-oxidant effects of ethanol. Furthermore, *S. cerevisiae* in active dry yeast form, used as starter in biotech and food industries, can suffer oxidative stress during biomass propagation and dehydration steps of their production, which could negatively affect yeast performance. Oxidative stress also influences the replicative lifespan of yeast, particularly important in re-pitching practices. Thus, protective treatments against oxidative damage with natural antioxidants, may have important biotechnological implications.

## Conclusion

MEL presents antioxidant properties in *S. cerevisiae*. However, these antioxidant properties were dependent on the dose and the phase at which stress was induced. In the absence of stress, MEL exposure appears to prepare cells for further oxidant assaults, whereby MEL clearly reduces oxidative damage in *S. cerevisiae* by decreasing ROS and oxidized glutathione (GSSG) levels. Our analysis offers insight into the effect of MEL on antioxidant defense systems in *S. cerevisiae*. However, the findings also raise a number of intriguing questions related to the regulation of gene expression by oxidative stress as a complex process controlled by different key regulators and intercommunication with different stress response pathways. Evaluation of the impact of MEL on transcription factors (especially Yap1p and Snk7p), or their influence on gene expression related to MEL receptors in *S. cerevisiae* could offer a productive avenue for further research. Furthermore, the effect of lower concentrations of MEL, closer to the ones found in fermented beverages, should also be assessed.

## Author Contributions

JV Design the experiments, perform and analyze the experiments, discussion of results and writing of the manuscript. BG and VS perform and analyze the experiments. AM, GB, and MT design the experiments, discussion of results and writing of the manuscript.

## Conflict of Interest Statement

The authors declare that the research was conducted in the absence of any commercial or financial relationships that could be construed as a potential conflict of interest.
